# Structural biology data archiving – where we are and what lies ahead

**DOI:** 10.1002/1873-3468.13086

**Published:** 2018-05-25

**Authors:** Gerard J. Kleywegt, Sameer Velankar, Ardan Patwardhan

**Affiliations:** ^1^ European Molecular Biology Laboratory (EMBL) European Bioinformatics Institute (EMBL‐EBI) Cambridge UK

**Keywords:** bioimaging, data archiving, structural biology

## Abstract

For almost 50 years, structural biology has endeavoured to conserve and share its experimental data and their interpretations (usually, atomistic models) through global public archives such as the Protein Data Bank, Electron Microscopy Data Bank and Biological Magnetic Resonance Data Bank (BMRB). These archives are treasure troves of freely accessible data that document our quest for molecular or atomic understanding of biological function and processes in health and disease. They have prepared the field to tackle new archiving challenges as more and more (combinations of) techniques are being utilized to elucidate structure at ever increasing length scales. Furthermore, the field has made substantial efforts to develop validation methods that help users to assess the reliability of structures and to identify the most appropriate data for their needs. In this Review, we present an overview of public data archives in structural biology and discuss the importance of validation for users and producers of structural data. Finally, we sketch our efforts to integrate structural data with bioimaging data and with other sources of biological data. This will make relevant structural information available and more easily discoverable for a wide range of scientists.

## Abbreviations

**3D**, three‐dimensional

**BMRB**, Biological Magnetic Resonance Data Bank

**CLEM**, Correlative Light and Electron Microscopy

**COD**, Crystallography Open Database

**cryo‐EM**, electron cryo‐microscopy

**cryo‐ET**, electron cryo‐tomography

**CSD**, Cambridge Structural Database

**EMDB**, Electron Microscopy Data Bank

**EMPIAR**, Electron Microscopy Public Image Archive

**IHM**, Integrative or Hybrid Modelling

**MSD**, Macromolecular Structure Database

**NDB**, Nucleic Acid Database

**NMR**, nuclear magnetic resonance spectroscopy

**PDB**, Protein Data Bank

**PDBe**, Protein Data Bank in Europe

**PDBj**, Protein Data Bank Japan

**RCSB**, Research Collaboratory for Structural Biology

**SASBDB**, Small‐Angle Scattering Biological Data Bank

**SEM**, scanning electron microscopy

**SXT**, Soft X‐ray Tomography

**wwPDB**, Worldwide Protein Data Bank

## Structural biology is well served by free and open archival resources

A cold winter's day in Stockholm, 10 December 1962 stands as one of the greatest landmarks in the history of structural biology (and of the MRC Laboratory of Molecular Biology in Cambridge). Kendrew and Perutz shared the Chemistry Nobel Prize (‘for their studies of the structures of globular proteins’) and Crick, Watson and Wilkins shared the Nobel Prize in Physiology or Medicine (‘for their discoveries concerning the molecular structure of nucleic acids and its significance for information transfer in living material’). The unravelling of the 3D structure of DNA and of the first globular proteins (haemoglobin and myoglobin) immediately impacted our understanding of biology. From then on, the history of structural biology has been a series of success stories that have shed light on countless biological processes, phenomena, mechanisms, etc., more than a few of which have been rewarded with Nobel Prizes themselves. Atomic models of anything from small peptides to huge assemblies such as ribosomes can now be determined routinely. In the first few decades, the advances were mainly on the molecular level, but recent progress in 3D bioimaging techniques (rewarded with Nobel Prizes for the development of super‐resolution microscopy and electron cryo‐microscopy) is extending the scope of (relatively) high‐resolution studies to complexes, molecular machines, organelles, cells and small samples.

Structural biologists study 3D structures ideally down to the level of individual atoms (the distance between two singly bonded carbon atoms is roughly 1.5 Å, where 1 Å is 10^−10^ m). The size of the objects that molecular structural biologists study varies from a few nm for small peptides, proteins, carbohydrates, RNA and DNA fragments, to over 100 nm for large viruses. Cellular structural biologists study cells (or parts or cells, or multicellular samples) at a scale about 1000 times greater than their molecular components. The biophysical techniques that molecular structural biologists use include crystallography (mostly using X‐rays as produced in powerful synchrotrons, but also neutrons and electrons), nuclear magnetic resonance spectroscopy (NMR), electron cryo‐microscopy (cryo‐EM) and electron cryo‐tomography (cryo‐ET). The latter two techniques, as well as others such as 3D Scanning Electron Microscopy (3D‐SEM), Soft X‐ray Tomography (SXT) and Correlative Light and Electron Microscopy, are used by cellular structural biologists.

The field of structural biology was very quick to understand the importance of a free exchange of data for the benefit of the entire community. A seminal meeting was held at Cold Spring Harbor Laboratory in 1971 which led to the establishment of the Protein Data Bank (PDB) in 1973 1, 2, 3. The PDB archived 3D models and data from (initially) crystallographic structural studies, and it is the oldest surviving molecular archive in biology. The PDB was a quintessential open‐access archive long before the term had even been coined. The models and data deposited in the 1970s can still be retrieved, used and viewed today, to the envy of many other communities.

Nowadays, the value of archiving and sharing research results (models, data, samples, etc.) is generally acknowledged and actively encouraged by most funding agencies and journals. The history of the PDB shows that what started as a way of sharing exciting new structures within a small community of fellow expert crystallographers has morphed into a resource that is used by numerous scientific communities. Few crystallographers would have predicted in the 1970s that their structures would see myriad uses far beyond answering the original research question which they were meant to address. Their structures can now be used to solve other structures, to produce models of structures whose experimental structure is not available yet, to design new or better low‐molecular weight ligands that might lead to new drugs, to simulate the dynamic behaviour and interactions of molecules and complexes, to compare a large number of related structures and investigate evolutionary relationships, etc. Furthermore, the availability of large numbers of models and the experimental data that underpins them has made it possible to develop methods to analyse models automatically (e.g. to find proteins with similar folds, to infer multimeric states, to predict active sites or interaction interfaces, etc.) and also to validate them.

Although in the past some structural biologists made their models available upon request (and sometimes not at all), it is now generally acknowledged that the best way to archive research outcomes (publications, models, experimental data) is in central archives that are operated by institutions or partnerships of institutions with a long track record of maintaining archives and attracting funding for this work. (A brief history of the change in mindset towards making deposition and release upon publication of coordinates mandatory can be found in 4.) The advantages of such centralized archiving are manifold and pertain to accessibility (one‐stop shops where all data in a certain domain can be found, with well‐defined formats and consistent annotation and validation), persistence (long‐term access to data is assured, and technology changes with respect to formats, hardware, or distribution methods are managed centrally and professionally) and context (archive‐wide searches, analyses, comparisons and ‘data mining’ are possible; the data are integrated with data from other relevant biological information resources). In addition, centralized archives also make economic sense as there is little or no duplication of effort (and funding). It has been estimated that the cost of archiving molecular structures in the PDB in perpetuity amounts to ~ 1% of the cost of determining a structure (labour, hardware, software, sample preparation, instrumentation, data collection, etc.).

The main current archives in molecular structural biology are the PDB 5 (which archives models and experimental crystallographic data as well as a subset of NMR data), BMRB 6 (which archives all NMR data), Electron Microscopy Data Bank (EMDB) 7 (which archives the volume maps derived from cryo‐EM and cryo‐ET experiments) and SASBDB 8 (which archives models and data from small‐angle scattering experiments). The main archives for cellular structural biology are EMDB (tomograms) and Electron Microscopy Public Image Archive (EMPIAR) 9 (raw data related to EMDB entries, as well as 3D reconstructions from other bioimaging techniques that are not supported by EMDB, such as 3D‐SEM and SXT). EMBL‐EBI 10 is directly involved in the running and operation of all these archives, with the exception of BMRB and SASBDB (with whom we collaborate). URLs for the websites of the archives mentioned here and in the following section can be found in Table 1.

**Table 1:** URLs for the major archives relevant to structural biology as mentioned in this Review. For more details, see the main text and the respective websites of these archives

Archive	URL
PDB	wwPDB: https://wwpdb.org/ with partner sites at: PDBe: https://pdbe.org/ PDBj: https://pdbj.org/ RCSB PDB: https://rcsb.org BMRB: http://www.bmrb.wisc.edu/
EMDB	https://emdb-empiar.org/
EMPIAR	https://empiar.org/
BMRB	http://www.bmrb.wisc.edu/
SASBDB	https://sasbdb.org/
PED^3^	http://pedb.vib.be/
IRRMC	https://proteindiffraction.org/
SBGrid Data Bank	https://data.sbgrid.org/
CXIDB	https://cxidb.org/
NDB	http://ndbserver.rutgers.edu/
CSD	https://www.ccdc.cam.ac.uk/
COD	https://crystallography.net/cod
PDB‐Dev	https://pdb-dev.wwpdb.org/

## Structural biology archives and what they have to offer

### Protein Data Bank

Work on establishing the PDB started in 1971 at Brookhaven National Laboratory (NY) 1. The first public release contained only a handful of structures, which were distributed upon request on magnetic tape. Later, CD‐ROM became the distribution medium of choice, but since the internet has become commonplace most structure downloads are taking place through ftp, web downloads or specialized APIs (application‐programming interfaces). The rise of the worldwide web and user‐friendly browsers in the early 1990s has been instrumental in making structure data accessible in an interactive fashion, with the first web‐based interface becoming available in 1995 11.

In 1998, the Research Collaboratory for Structural Biology (RCSB) 12 took over the responsibility of running the PDB. Two years earlier, the Macromolecular Structure Database (MSD) had been established at a then still very young EMBL‐EBI. To ensure that there would only be one single worldwide archive for macromolecular structures, RCSB, MSD (nowadays called PDBe 13) and PDBj 14 came together in 2003 to establish the Worldwide PDB (wwPDB) organization which has since then shared the responsibility of managing the PDB archive (BMRB 6 joined in 2006) 15.

The wwPDB partners, who are independently funded, collaborate on all aspects of the PDB archive (e.g. deposition requirements, release policies, annotation procedures, validation standards, description of ligands, interactions with structure‐producing communities and journals). The only exception is the so‐called ‘data‐out’ side, i.e. the ways in which the PDB data (which is identical at all sites) are made available to users. This includes their websites, specific tools or services, enrichment of PDB entries with other data, helpdesk, outreach, training and public‐engagement activities. The partners therefore each have their own website, with unique features, tools and services, and are engaged in ‘friendly competition’ to attract users. The users benefit from the competitive aspect which encourages the sites to offer new and improved services, and they ‘vote’ with their mouse clicks. The wwPDB partners together support hundreds of millions of structure downloads every year.

The wwPDB partners accept depositions of new structures from their own geographical area (for instance, PDBe is responsible for structures from Europe and Africa). The sites use a common software system, OneDep 16, for the deposition, validation and annotation of the depositions 17. After approval by the depositors, the processed entries are either released immediately (on a weekly cycle), or withheld until publication or for a maximum period of 1 year. In 1993, the 1000th PDB entry was released, and in 1999 the 10 000th. In recent years, the archive has grown by around 10 000 entries per year. On 14 May 2014, the PDB contained over 100 000 entries for the first time 18, and in May 2018 the archive reached the 140 000‐entry point.

### Electron Microscopy Data Bank

In 2002, long before cryo‐EM became a major structure‐determination method (and was often irreverently referred to as ‘blobology’), a European initiative led to the establishment of the EMDB 7, hosted, run and maintained by EMBL‐EBI. EMDB archives 3D volume maps from cryo‐EM and cryo‐ET studies (any atomic models based on such studies continue to be archived in the PDB). In 2007, RCSB PDB joined this effort and the two partners shared the responsibility of developing the resource further, integrating the EMDB deposition system with that of the PDB, and annotating the depositions. With the full integration of the EMDB and wwPDB deposition, validation and annotation systems in OneDep, PDBj joined the franchise as a deposition and annotation site for Asia.

**Figure 1:**
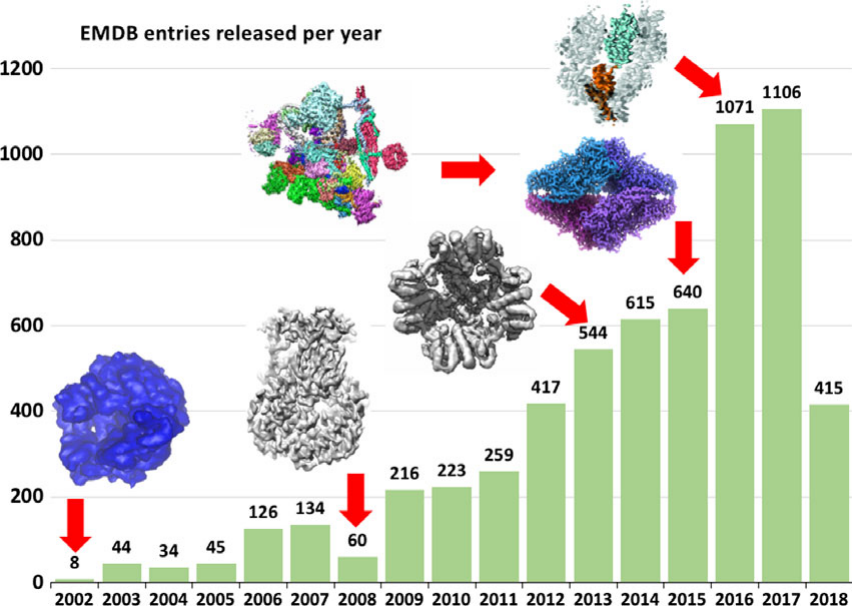
The number of EMDB entries released per calendar year since its inception in 2002 (up‐to‐date statistics and graphs can be obtained from https://emdb-empiar.org/emstats). A few structures are highlighted for some of the years: 2002: EMD‐1003 59, the oldest surviving single‐particle structure in EMDB, of a 70S *Escherichia coli* ribosome, released on 22 August 2002; 2008: EMD‐1461 60, the first structure (of VP6) with a claimed resolution below 4 Å, before there were direct electron detectors; 2013: EMD‐5778 61, the first TRPV1 structure from Yifan Cheng's laboratory showing that EM could be used to get to sub‐4 Å structures of biologically important proteins; 2015 (multicoloured): EMD‐6413 62, the first structure of the spliceosome, an exceptionally challenging complex, at high resolution; 2015 (blue‐purple): EMDB‐2984 63, first very high resolution single‐particle structure, of beta‐galactosidase (2.2 Å); 2016: EMD‐4015 64, a subtomogram averaged structure of HIV‐1 capsid‐SP1 at 3.9 Å from John Briggs’ laboratory. To get more information about an EMDB entry, e.g. EMD‐1003, visit https://emdb-empiar.org/emd-1003.

Starting out with eight maps in 2002, EMDB reached the 1000‐entry milestone in 2011, and currently holds over 6000 maps 19, Fig. 1. About 6% of the EMDB entries are full tomograms, and another 10% are so‐called subtomogram averages (maps for a specific molecule or complex that have been obtained by averaging multiple copies of that complex encountered in a full tomogram, which often makes it possible to produce an atomic model of it). Three quarters of all entries have been determined by the most popular cryo‐EM technique, single‐particle EM. It is in the latter area that the spectacular increases in resolution have taken place in the past few years 20. From 2002 until 2015, the average resolution of single‐particle maps deposited to EMDB fluctuated around 15–20 Å. In the years since then, this number has come down to under 8 Å, and in 2017 as many as 315 entries had a resolution better than 4 Å, representing 28% of all EMDB entries released that year. The average resolution of tomograms in EMDB has generally been in the 25–75 Å region, and for subtomogram averages around 30 Å. However, with the latter technique resolutions better than 4 Å have also been achieved in recent years.

### Electron Microscopy Public Image Archive

**Figure 2:**
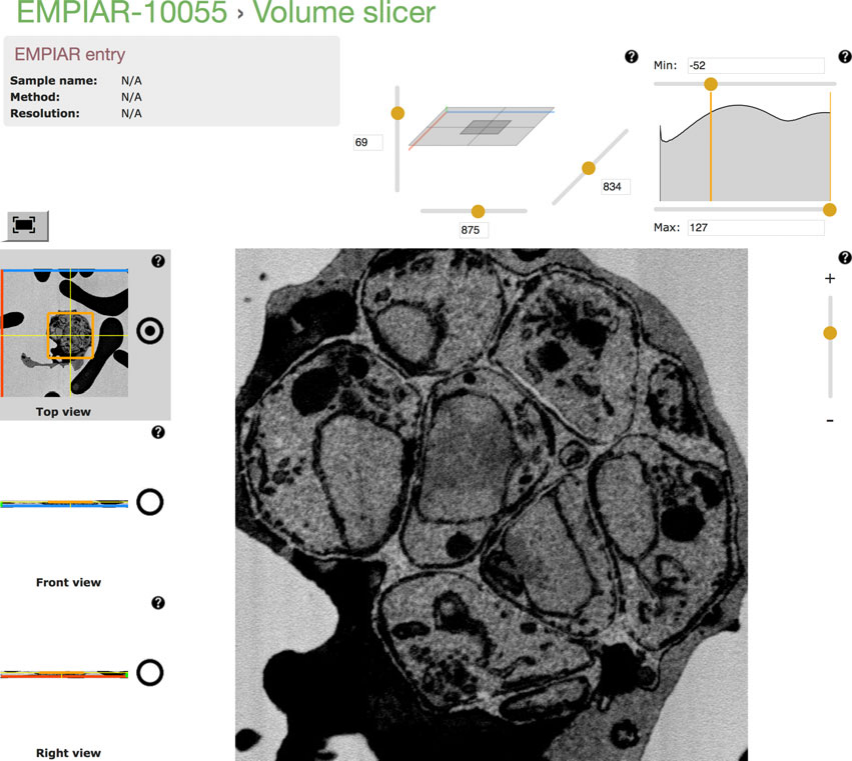
EMPIAR entry 10055 65 is a 3D EM map obtained with one of several techniques not currently supported by EMDB (in this case, Serial Block Face Scanning EM). Using the interactive volume slicer, 2D slices through the 3D volume can be inspected without any need to download the data or any software explicitly. The URL for this page is: https://www.ebi.ac.uk/pdbe/emdb/3dbrowse/empiar_10055_l-schizont_2.

After consultations with the molecular and cellular 3D bioimaging community in a series of workshops 21, a strong recommendation from the field was to establish an archive where the raw experimental data from cryo‐EM (mostly 2D images and movies) could be stored. In response to this, the EMPIAR archive was established at EMBL‐EBI in 2014 9. The archive's scope initially was to enable deposition of the raw data underpinning studies that had led to one or more map depositions in EMDB, and this continues to be the case for the majority of EMPIAR entries. However, nowadays the archive also accepts depositions of data from 3D‐SEM and SXT experiments, which cannot be accommodated in EMDB. Having started out with 16 entries in 2014, EMPIAR currently has over 150 released entries, the largest of which takes up over 12 TB of disk space. The data in EMPIAR are used for a variety of purposes including validation and re‐determination of published structures, development and testing of new software and validation methods, education and training, and community challenges (such as the Map Validation Challenge organized by the EMDataBank project partners). EMPIAR data can be downloaded in a variety of ways, and some of the raw data frames can be viewed directly in a web browser on the EMPIAR website. For some of the entries that are not related to an EMDB entry, a 3D volume map is available through EMPIAR and can be inspected interactively using a 3D volume slicer 22, Fig. 2.

### Related archives

There are a number of other archives that hold 3D models or data of molecules or complexes that are of biological interest. The Biological Magnetic Resonance Data Bank (BMRB) is a repository of experimental NMR data 6. These are mainly assigned chemical shifts, but also include experimental restraints, relaxation parameters, spectral peak lists, metabolomics data and raw spectral data. BMRB currently contains over 12 000 entries. The chemical shift and restraint data pertaining to structures in the PDB are also distributed by wwPDB.

The Small‐Angle Scattering Biological Data Bank (SASBDB) was established at EMBL‐Hamburg in 2014 8. It accepts SAXS, SANS and WAXS data of biological macromolecules and complexes, nonatomistic (‘bead’) models and atomistic models that have been determined solely using SAS methods. If SAS is used as a supporting technique for structure determination by X‐ray, NMR or cryo‐EM, the models are archived in the PDB and the SAS data can be deposited to SASBDB as part of a OneDep deposition session. SASBDB currently contains almost 600 experimental datasets and over 900 models.

A resource that is somewhat related to the previous two is PED^3^, the Protein Ensemble Database 23, which holds ensembles of structural models of intrinsically disordered proteins based on NMR and/or SAXS data. It has 24 entries (encompassing over 25 000 individual models) but this number has been constant for some time as data submissions have been suspended since January 2016.

There are several archives where raw X‐ray diffraction data can be deposited nowadays, including IRRMC 24 (Integrated Resource for Reproducibility in Macromolecular Crystallography; currently holding ~ 6400 entries), SBGrid Data Bank ( https://data.sbgrid.org/; over 400 datasets), and CXIDB 25 (Coherent X‐ray Imaging Data Bank; over 70 datasets).

The Nucleic Acid Database 26 (NDB) was founded at Rutgers University in 1995 and contains almost 9400 3D structures, most of which are also in the PDB. For crystal structures of small molecules, some of which are of biological interest, two main repositories are the Cambridge Structural Database (CSD) 27 and the Crystallography Open Database (COD) 28. CSD has been around since 1965 and currently contains over 900 000 curated entries. COD is a more recent initiative and contains over 390 000 entries.

### Downstream resources

The availability of many thousands of 3D structures of biomacromolecules and their complexes, combined with the importance of structural information for understanding, and potentially modifying, the function, activity, specificity, interactions, etc. of such entities has stimulated the development of hundreds of downstream resources. Such resources may provide a different view of the data held in the archive (for instance, with added annotation, visualizations or validation information), they may combine structural information for a particular class of molecules (kinases, viruses, antibodies, RNA, etc.) with data from other resources (sequences, activity, etc.), or they may focus on a particular aspect of structure across the archive (domain definitions, ligands, active sites, surface properties, etc.). The journal *Nucleic Acids Research* publishes a special Database issue once a year, with descriptions of new bioinformatics resources and updates about existing ones 29, and it also maintains an online molecular biology database collection ( http://www.oxfordjournals.org/nar/database/c/). At present, over 200 downstream resources use PDB data 12. Besides research‐oriented databases, there are also educational resources for both students and scientists who are not specialists in structural biology, such as PDB‐101 12 and Proteopedia 30.

### Future resources

Until recently, the world of structural biology data archiving was fairly simple: there were three main techniques (X‐ray, NMR, cryo‐EM), three major archives (PDB, EMDB, BMRB), the systems studied varied in size from small peptides to ribosomes and viruses, and all models that resulted from interpretation of the experiments were atomistic (although the data itself rarely resolved individual atoms). In recent years, this picture has been changing as methods and ambitions have evolved. The ambition to elucidate the structure of ever bigger and more complex systems, on scales from atoms to cells, has led structural biologists to employ a much wider portfolio of biophysical and other techniques that provide lots, or perhaps just snippets, of structural information (e.g. SAXS/SANS, FRET, mass spectrometry). This heterogeneous set of methods, with variable information content, may deliver models with atomistic detail, or perhaps detail at the level of residues or secondary‐structure elements, or even of domains or entire molecules. In the latter cases, atomistic models are generally not an appropriate representation, so that bead models (e.g. from SAXS experiments), segmentations (e.g. of cryo‐EM maps), envelopes or geometric shapes are produced instead. When data from multiple experiments are used, the final model may be a colourful mix of atomistic and ‘blobby’ components, perhaps with the odd theoretical model thrown in for good measure. This way of piecing together models from multiple heterogeneous data sources is referred to as Integrative or Hybrid Modelling (IHM) 31.

The PDB is currently not set up to deal with heterogeneous collections of models and data. For this reason, a task force was established and following its first meeting in 2014 a white paper was produced giving recommendations on how IHM results should be archived 32. The development of standards for model representation, validation, visualization and publication was one top priority that emerged, along with the need for a federated system of archives across which heterogeneous models and data can be stored. Members of the task force and the wwPDB partners have developed PDB‐Dev 33, a prototype deposition system for IHM models. To fully support archiving of heterogeneous data, suitable method‐specific archives will have to be identified or developed for the types of data used in IHM studies (FRET, MS, DEER, EPR, AFM, etc.) and become part of the biomacromolecular structure‐archival flora and fauna.

## Validation of structural data and models is (still) necessary

### Why is validation necessary?

In the past half‐century, structural biologists have produced an astonishing array of beautiful structures that have provided new insights into biology and fundamental processes involving health and disease. However, much as the animals in Orwell's ‘Animal Farm’ are all equal, but some are more equal than others, some structures are more ‘correct’ than others. The fundamental cause of this is that all structures are really models: a structural biologist's best effort to faithfully interpret and represent the experimental data 34. Experimental data by its nature can be noisy, incomplete and contain errors, all of which will complicate its interpretation. In the case of X‐ray crystallography, for instance, the data are space‐averaged over all the molecules in the crystal, and time‐averaged over the duration of the diffraction experiment. The collected data may be weak or incomplete, have a low degree of redundancy (i.e. not many independent observations of the same reflections), will only extend to a certain resolution which in most cases is not sufficient to resolve individual atoms, and that's just the diffraction intensities. In addition, the unknown phases have to be retrieved somehow and they will contain errors as well.

Moreover, not every structural biologist is equally experienced and skilled. Experience comes into play when subjective decisions need to be made in the model‐building and refinement process (even at atomic resolution). The structural biologist needs to be almost a Renaissance person and have a good knowledge and understanding of the history, composition and biology of the sample, of chemistry and physics, of crystallography and space groups, and of all the aspects of the structure‐determination process, from sample to final model. When the data are of good quality and high resolution and the crystallographer is skilled and experienced, the final model is unlikely to have major issues. When either, but not both, of these conditions are fulfilled, it is quite possible that the final model will have no major issues. However, when there are problems with the data and the crystallographer lacks experience or skills, one can only hope that the final model will have no major issues. Modern model‐building and refinement software makes it more difficult to make mistakes, but even if such software is largely fool‐proof, it is certainly not damn‐fool‐proof.

Mistakes that crystallographers have made range in severity 35, 36: in a few known cases, entire proteins or domains have been built incorrectly (e.g. with the directionality of the chain trace reversed, so that the C terminus was built where the N terminus ought to be etc.). Sometimes, the mistakes have been limited to one or a few secondary‐structure elements. Errors in modelling flexible regions are not uncommon as are mistakes in building the main chain or sidechain conformation of individual amino‐acid residues. Quite common are issues with small‐molecule ligands where mistakes may happen in determining their identity, placement, orientation and conformation. Since ligands by their nature are infinitely more varied than the standard amino acids and nucleotides, they are more difficult to build, refine and validate 37, 38, 39, 40, 41.

### What is validation?

Model validation is the use of statistical and other techniques to assess the quality and reliability of a model, both on its own and in light of the underpinning experimental data. Basic questions that a structural biologist should address before even thinking about writing a paper or depositing a model include:

- Does the model explain all the experimental data that were used?
- Does it explain all the prior knowledge that was used?

However, these questions only test whether the model‐building, refinement and other procedures have done their job properly, given the information that was available. Much more important are questions that aim to find independent (‘orthogonal’) supportive evidence that the model is reliable and has predictive value, such as:

- Does it explain any experimental data that were not used?
- Does it explain any prior knowledge that was not used?
- Is the model the best possible, most parsimonious explanation of the data?
- Are testable predictions based on the model correct?

Nonaffirmative answers to any of these questions should make the structural biologist go back to the data and see if the model contains errors or could otherwise be improved.

In most cases, structural biologists will have much more experimental data available than just, say, the crystallographic data collected on their sample. For instance, there may be binding data available for relevant small molecules, there may be information about the behaviour of mutants, sequence comparisons may reveal conserved residues that may be important for structure or function, etc. There may also be information about the structure from other biophysical methods, e.g. the overall shape of a molecule or complex from a SAXS experiment. Finally, a structural biologist may ‘hide’ some of the experimental data and use it only to test how well the model fits this ‘unknown’ data. In X‐ray crystallography, this can be done by setting apart 5–10% of the data and calculating a ‘free’ R‐value during the structure‐determination process without ever using that small subset of data in refinement. This process is known as cross‐validation: the conventional R‐value measures how well the model explains the data, but the model can easily be adjusted to fit the data better, without being correct (this is called over‐fitting). The free R‐value cannot be ‘fudged’ in this way and thus provides a better measure of how well the model predicts the data. In cryo‐EM, people can take an even more drastic approach and split the data into two equal‐sized ‘pots’ and produce an independent model for each of these. If the models truly explain the signal in the data (which should be the same in both cases) but not the noise, they should fit the other dataset (almost) as well as their own dataset. In general, if the model fits experimental data that were not used in its construction, this is a very encouraging indication that the model is reliable.

Structural biologists usually also have different sources of prior knowledge about the chemistry and physics of the kind of molecules they have in their sample. Some of this knowledge can be used during the structure‐determination process, e.g. knowledge of ideal bond lengths and angles, knowledge of the stereo‐chemistry of the molecules, the amino‐acid or nucleotide sequences of the molecules in the sample, the fact that nonbonded atoms cannot approach each other too closely, the chemical structures of any ligands, and knowledge of biosynthetic pathways of carbohydrate decorations on proteins, which determine which type of monomers can and cannot be expected in such glycosylation sites. There is usually also prior knowledge that is not used directly, e.g. about the expected distributions of phi/psi torsion‐angle values for proteins (Ramachandran analysis), about energetically preferred conformations of amino‐acid sidechains (rotamer analysis), about preferred or unlikely interactions and environments (e.g. of charged residues), etc. Again, if the model fits expectations based on prior knowledge that was not used directly, this reinforces the expectation that the model may be reliable.

### Trust but verify. How can I do that?

Fortunately, users of the PDB do not have to be expert structural biologists themselves to assess the quality and reliability of models they retrieve from the archive, or even to select the most suitable from a number of alternative models for the same molecule or complex. In consultation with community experts 42, 43, 44, wwPDB and EMDB have produced a software pipeline for the validation of structures determined by crystallography, NMR and cryo‐EM 45. The validation process assesses each model in the light of the experimental data and prior knowledge to find out:

- if it reproduces the data/information/knowledge used in the construction of the model (e.g. looking at the conventional R‐value for X‐ray structures, checking the bond lengths and angles, checking the chirality, looking for unusually short distances between nonbonded atoms, etc.);
- if it predicts any data/information/knowledge that was not used in the construction of the model (e.g. the free R‐value, the number of outliers in the Ramachandran analysis, unusual sidechain conformations, etc.).

Some of the validation criteria report on local properties (e.g. atoms that are too close), some on global properties (e.g. the R‐value), and some on both (e.g. individual Ramachandran outliers as well as overall Ramachandran‐plot statistics). Some of the validation methods only consider the model, a few of them only consider the data, and another few consider the fit of the model to the data.

The results of the validation analysis are collected in a report that is human‐readable, as well in a file that is more suitable for further utilization by software. The validation report contains a one‐page summary that should be useful to editors, reviewers and users who are not themselves structural biologists. The rest of the report provides more detailed analysis of the data, the composition of the model, a summary of the quality of each molecule (both overall and on a residue‐by‐residue basis) and finally the summarized results of many individual validation checks (bond lengths, Ramachandran, fit of model to the density for crystal structures, etc.).

One important issue to keep in mind is that any outliers listed in the validation report of a structure are not necessarily mistakes. In general, outliers can be one of two things: a genuine, albeit unusual feature of the structure, or an error in the model. Often the only way to be sure is to check the outlier in the light of the experimental data. In the case of crystal structures, this can be done by checking the electron‐density maps around the outlier atoms, residue(s) or ligand. If the density is clear and convincing, the outlier is most likely a genuine but unusual feature of the structure (and structural biologists might well want to describe such unusual features in their publication of the structure). If, on the other hand, there is no or very poor density, or the density is good but the model does not fit it very well, the outlier is more likely to be an error in the model (which a structural biologist would want to try and fix). It is always good to keep in mind the following adage: extraordinary claims require extraordinary evidence. For structural biology, this means that one should be extremely hesitant to formulate (or, as a reviewer, reader or user, to accept) hypotheses or interpretations or inferences based on parts or aspects of a model that are not extremely well defined and supported by the experimental data.

The validation reports are available from the sites of all wwPDB partners, and these sites also offer tools to inspect the electron density for crystal structures in the PDB. The PDBe website even makes it possible to sort search results based on a combined criterion that aims to reflect the overall quality of PDB entries. If a search results in more than a few hits in the PDB, this can be helpful in narrowing the hits down to a manageable number. Of course, when searching for the most suitable PDB entry for a particular purpose, many other factors can come into play (taxonomy, sequence variants, completeness of the model, presence of a particular ligand, experimental conditions, etc.). Nevertheless, sorting by quality always helps in identifying any less reliable models using more sophisticated criteria than just the resolution and R‐value.

At present, validation methods for X‐ray structures (especially of proteins) are well established and there is community consensus about them. Developing methods to validate structures determined by NMR and cryo‐EM is still very much an active research topic. Hopefully, these methods will reach the same level of acceptance and consensus as X‐ray validation methods in the next 5 years or so, leading to better validation reports for such structures and better validation information to help users make informed choices when searching and selecting structural data in the PDB or EMDB.

## The future is bright and multiscalar

### Integrating structure and 3D bioimaging data

Until fairly recently, the focus of the field of structural biology was largely molecular. Symmetrical molecular assemblies such as viruses were exceptions, because the high symmetry made it possible to resolve the individual coat components to reasonably high resolution, allowing reconstruction of the entire coat by application of symmetry operators. In the mid‐1990s, the first complex ‘molecular machines’ yielded to crystallographic structure determination (e.g. the GroEL‐GroES complex, the nucleosome), and in the year 2000, the first ribosome structures were reported. With the rapid advances in optical and electron microscopy, the range of length scales at which biological structures can be studied in detail and in three dimensions is expanding, and the gap between electron and light microscopy is being bridged. Nowadays, cellular structural biology enables the study of biological macromolecular assemblies, complexes and machines (previously studied with molecular structural techniques) in the context of the cell 21. Conversely, it offers cell biologists access to atomistic models of (components of) systems they are interested in. To provide links between structural information at a range of scales, a number of requirements need to be fulfilled. First, both the cellular and the molecular data need to be publicly available. Second, the contents of the cellular bioimaging datasets have to be captured and identified in a way that is both meaningful for humans and amenable to coupling with the contents of bioinformatics resources. Third, there is a need for software with which all the available structural data, at a variety of length scales, can be visualized, analysed and traced to other resources of biological sequence, structure and function data.

**Figure 3:**
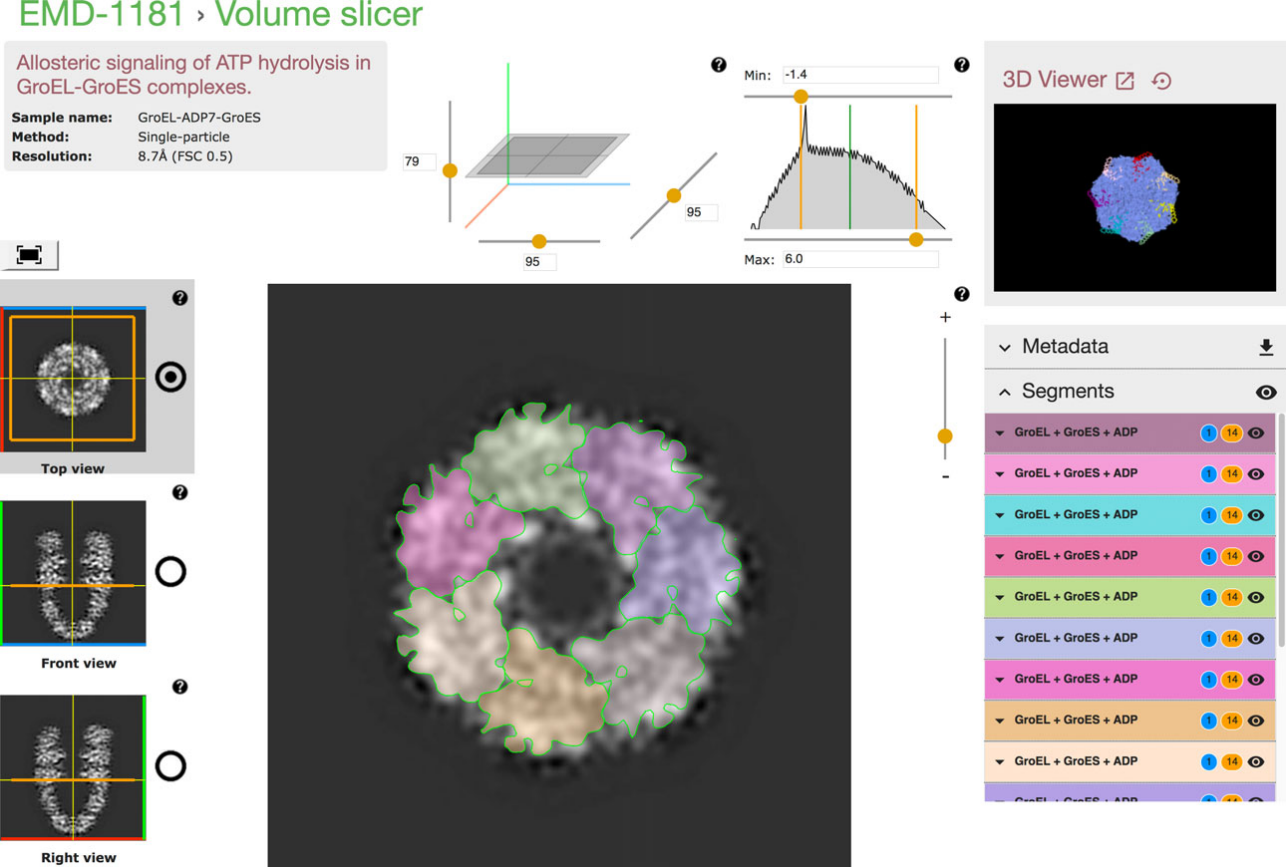
Prototype version of the volume browser that is being developed at EMBL‐EBI. Here, the basic functionality is demonstrated on a single‐particle volume map of a molecular machine, the GroEL‐GroES complex. The volume slicer allows 2D slices to be displayed with the identified segments superimposed, each in a different colour. The panel on the bottom right shows some of the ontological terms associated with the segments, in panes of the same colour as the corresponding segments in the slice viewer. The smaller 3D viewer in the top right shows the entire volume map (EMDB entry EMD‐1181) and the fitted atomic model (PDB entry http://www.rcsb.org/pdb/search/structidSearch.do?structureId=2C7D) 66. We envisage that this way of visualizing structural data will become particularly powerful when cell‐scale imaging datasets are segmented and annotated.

Established archives such as PDB, EMDB and EMPIAR, as well as emerging archives that cover light‐microscopy data, fulfil the first requirement. The second issue is addressed by so‐called segmentation of 3D volume data, where shapes that correspond to individual organelles, assemblies or molecules are delineated in three dimensions, usually through a combination of automatic and manual work. Often, these segmentations (commonly referred to as regions‐of‐interest or ROIs, in light microscopy) are also identifiable (e.g. as microtubules, polysomes, nuclear pore complexes or components of a ribosome). If the segmentations can be linked to objects or concepts or entries in other biological data resources (e.g. UniProt 46, Gene Ontology 47, EMDB or PDB), they become identifiable in information searches and discoverable through links to and from such resources. At EMBL‐EBI, we are working on ways to make it easy to carry out this step. To this end, we are developing a format (EMDB‐SFF, for Segmentation File Format) to represent segmentations and their biological annotation, building on the outcome of a workshop with community experts 48. In addition, we have developed a web‐based tool to carry out the annotation and to save the results in an EMDB‐SFF file, which can be uploaded when the data are deposited to EMDB or EMPIAR. We are also developing a web‐based volume browser which will make it possible to visualize the relevant 3D information (bioimaging data, segmentations, relevant EMDB maps and PDB structures) in one interface, Fig. 3.

### Integrating structure and other biological data

Integration of structural and other data is not only important for 3D segmentations, but also for atomistic models, to improve the biological data ecosystem and thereby make structural data more easily discoverable and accessible. In 2002, UniProt and PDBe started a project to link protein structures in the PDB to other resources related to sequence, function and taxonomy (SIFTS 49). The basis of this is the identification of the appropriate UniProt entry for any protein molecule in the PDB, and then mapping the residue‐level correspondence between the sequence and the structure. Other resources to which the PDB proteins are linked include Pfam 50, CATH 51, SCOP 52, GO, InterPro 53 and the NCBI Taxonomy resource 54. In recent years, the protein links from PDB to UniProt have been improved to include mapping to the correct isoform and mapping to proteins with similar sequences (at least 90% sequence identity). SIFTS has become the *de facto* standard for protein sequence‐to‐structure linking and is used by many other bioinformatics resources. In a related effort, the Rfam 55 and PDBe teams have recently joined forces to provide RNA‐family information for all RNA molecules in the PDB (at present, about 3800 PDB entries contain a total of ~ 10 000 RNA molecules). This information will make it easier to search for and identify PDB entries containing RNA.

FunPDBe is a new project, carried out in collaboration with a number of structural bioinformatics groups that aims to provide detailed functional annotation of proteins (and individual residues) in the PDB. The partners will develop data standards to represent these functional annotations and implement an integrated resource for data delivery. Web components to display annotations will be developed and will allow easy access to these data by the wider community. In the first instance, the project will focus on annotations related to functional sites (both manually annotated and predicted), biological assemblies, enzyme catalytic sites as well as the effects of genetic variants and mutations on structure and function.

### Delivering appropriate structural data to users like you

Efforts such as SIFTS and annotation of segmented EM datasets help make structural data discoverable by providing links between structural archives and other biological data resources. In this way, such resources can provide links to appropriate PDB or EMDB entries, or they may provide structure‐visualization tools that show the biological annotation (e.g. about disease‐linked mutations or sequence domains) directly in the context of the 3D structure. We also provide our own set of visualization tools to help our users to visualize and analyse 3D structures, as well as to study biological annotation (e.g. secondary structure or catalytic residues) in the context of the structure. The volume browser discussed earlier is a good example of this, where experimental bioimaging data, annotated segmentations and relevant PDB or EMDB structures are all presented in a single interface, Fig. 3.

**Figure 4:**
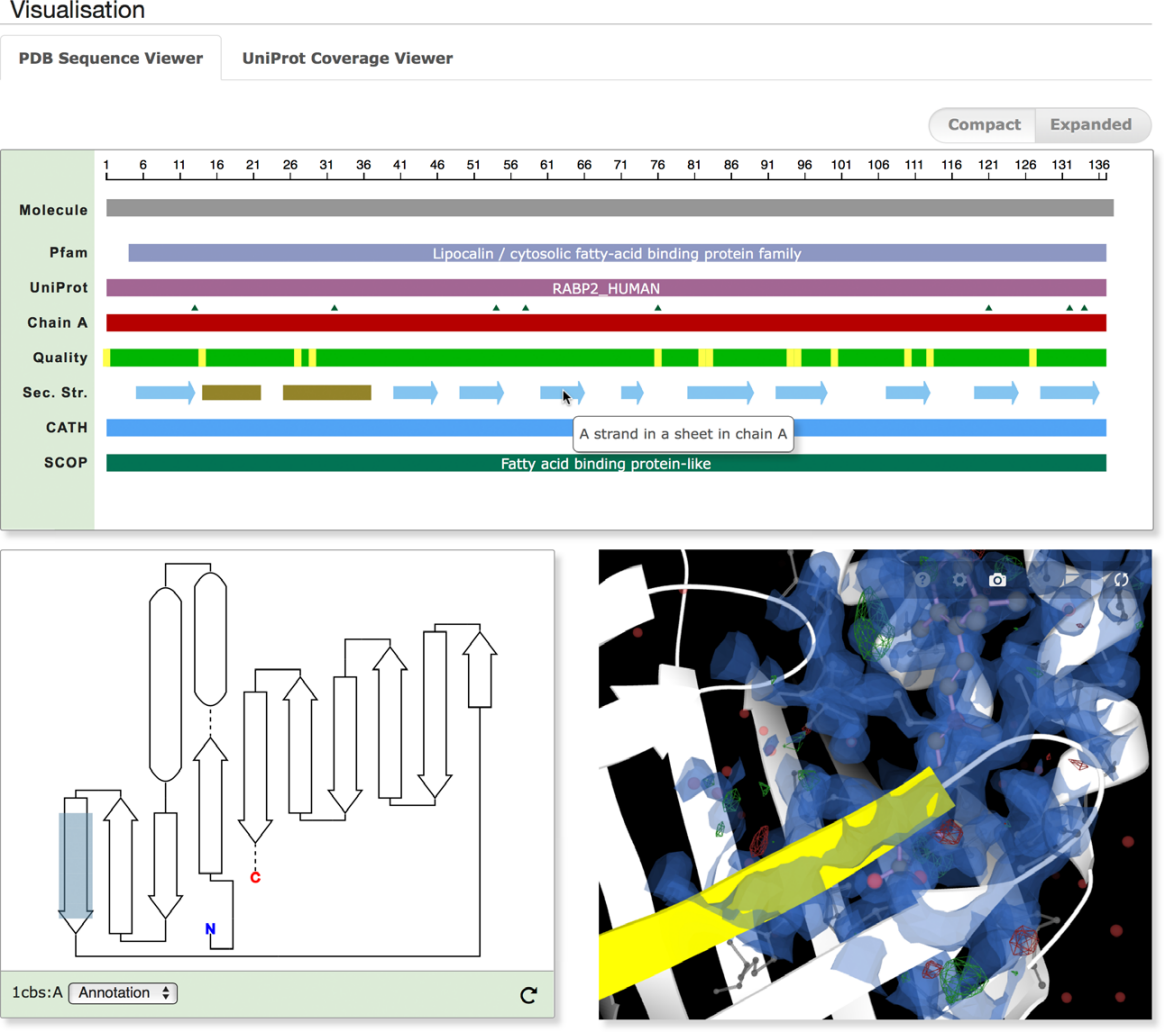
Examples of the coupled sequence, topology and 3D displays that PDBe offers for all proteins in a PDB entry, in this case human cellular retinoic‐acid‐binding protein type II in entry http://www.rcsb.org/pdb/search/structidSearch.do?structureId=1CBS 67. The cursor points at one of the secondary‐structure elements in the sequence display (in this case a beta‐strand). The same strand is automatically highlighted in blue in the topology diagram and in yellow in the 3D display. Note that all three displays are interactive and that the 3D viewer also enables the user to inspect any underlying X‐ray or EM volume map 68. The URL for this page is: https://www.ebi.ac.uk/pdbe/entry/pdb/1cbs/protein/1.

Thanks to resources such as SIFTS, there is a lot of biological annotation available for proteins in the PDB. Although much of the information is in the form of annotations at the level of the sequence (i.e. one‐dimensional), it is often very helpful to inspect it in higher dimensions, as this helps one's understanding of both the structure itself and the context of the annotated features in the structure. For every protein molecule in the PDB, we provide a linked 1D‐2D‐3D display of the protein sequence, structure and annotated features, Fig. 4. The 1D display is simply the amino‐acid sequence with extra bands of annotation to identify secondary‐structure elements, sequence domains, ligand‐binding residues, residues with a poor fit to the experimental data, etc. The 2D display is a flattened representation of the topology of the protein, i.e. displaying the organization, directionality and connectivity of all secondary‐structure elements and their nearest neighbours. This is a useful intermediate level for studying protein structure between the 1D sequence and the full spatial arrangement of all atoms. Finally, there is a viewer that shows the 3D structure, either in full, or zoomed to reveal local details. The information in all three displays is coupled, so for instance clicking on an active‐site residue in the 1D sequence display will highlight the location of that residue in both the topology diagram and the 3D display, and clicking on a helix in the topology diagram will reveal where it lies in both the sequence and the 3D structure.

Given the importance of the validation of structural data before use, we have prioritized the development of tools to help users (who may not be specialists in, say, X‐ray crystallography) do just that. The 3D structure viewer for PDB entries can display the supporting experimental maps for crystallography and cryo‐EM structures. For every ligand in a PDB structure, there is a separate display of each instance of the ligand, its surrounding neighbours and the local maps. This greatly facilitates the assessment of whether or not the ligand's presence is supported by the experimental data and if its positioning, orientation and conformation make sense in light of the data and its environment. It is well known that even today there are often issues with the reliability of ligands modelled inside structures deposited in the PDB, so such tools are tremendously useful. For EMDB entries, there are also a variety of interactive and static displays of information that have been designed to help users assess their quality as well as the quality of the corresponding fitted PDB model (if there is one) 56.

All the tools described in this section have in common that they do not require the installation of any software: they will work in any reasonably modern web browser on devices ranging from mobile phones to high‐end graphics displays. Nor is there a need to download any data in advance (such as PDB models or EMDB maps), as all data are retrieved by the tools as and when needed. All the tools (and the archived data) are free to use, and some of them can even be included in external websites. For advanced users, we also offer APIs through which structures and annotations can be accessed programmatically.

## Conclusions and perspectives

There are a number of challenges that need to be (or are being) addressed by the structure‐archival community, including:

- How to deal with the increasing number, size and complexity of deposited structures and experimental datasets;
- How to deal with multiple heterogeneous sources of structural information at a range of length scales;
- How to coordinate efforts across multiple disciplines that produce structural data to ensure that the data can be archived, exchanged, integrated and disseminated efficiently;
- How to integrate structural data on scales from atoms to cells;
- How to integrate structural information with other sources of biological, chemical, and potentially medical data;
- How to deliver appropriate structural data to nonspecialists, ideally in the context of their work.

Our Review has highlighted a number of ongoing initiatives, both at EMBL‐EBI and in a global context, to address these challenges. Ten years from now, more and more requests for structural data are likely to originate from other resources that present their own data in the context of 3D structural data. Similarly, instead of accessing a single structure, users will be offered views of the context of that structure (e.g. as part of a complex, or as a component of a specific enzymatic pathway, or inside its cellular environment).

Structure deposition is now mandatory for most journals and funders, and if a model is deposited into the PDB the supporting experimental data must also be deposited. Nevertheless, there remains a substantial amount of ‘black matter’, i.e. structures that have not been deposited. For legacy structures, if the experimental data are still available, these can still be deposited to the PDB. Legacy cryo‐EM maps can be deposited to EMDB, as can the full image datasets that underpin them (to EMPIAR). One significant (and continuing) source of unpublished structural data is industry, especially in pharma, agriculture and biotech. The typically large numbers of structures determined in traditional structure‐supported design efforts and in fragment‐screening exercises provide a treasure‐trove of information that improves our understanding of protein–ligand interactions and can be used to improve algorithms for virtual screening and ligand‐protein docking. Some companies 57 and academic groups 58 have already made commendable efforts to deposit substantial sets of structures of the same protein in complex with different ligands or fragments, and we hope that many more will follow their example.
